# The Tuberculosis Problem in Buffalo1Read at the Buffalo Academy of Medicine, January 12, 1909.

**Published:** 1909-03

**Authors:** John H. Pryor

**Affiliations:** Buffalo, N. Y.


BUFFALO MEDICAL JOURNAL.

Vol. Lxiv. MARCH, 1909. No. 8

### ORIGINAL COMMUNICATIONS.

### The Tuberculosis Problem in Buffalo[1]

By JOHN H. PRYOR, M. D., Buffalo, N. Y.

A quarter of a century has passed since the medical profession generally accepted the belief that tuberculosis is a curable, preventable and unnecessary disease. Recently the public has been sedulously taught these facts in every state, city and hamlet. Now the people have begun to ask awkward questions. They want to know how much this vaunted knowledge has benefited the mass of consumptives, and the honest answer is very little. They are inquiring insistently why the disease prevails so mercilessly, and why the appalling death rate with its awful results continues almost unabated? This is a tremendously encouraging sign. Education is at last arousing the public and the day of a new form of enlightened endeavor is near at hand.

At the present time there exists a strong probability that the public will assume the great task of checking, and gradually annihilating a communicable disease and its dire effects. There is a prospect that the medical profession will be compelled to perform its full duty or be relieved of its plain responsibility. The burning question today is this, shall the medical profession lead as of old in the crusade against disease or follow in the titanic struggle? For the demand that the frightful suffering, woe and devastation shall cease, and the righteous clamor for adequate protection from an uncontrolled enemy of mankind, will be irresistible in its gathering force. As yet the whole truth has not been told. The grim story of disgraceful neglect and apathy, and the consequent unparalleled hardship of the poor consumptive will sometime be recited in fitting language by the men who have grown ugly while they view the Carnival of Misery and listen to the unnecessary death rattle in the weary victim's throat.

The tuberculosis problem in Buffalo is somewhat dissimilar to that existing in other large cities. There are reasons why the disease can be attacked with greater promise of success than in

1. Read at the Buffalo Academy of Medicine, January 12, 1909.

most large centers of population. The death rate from tuberculosis is comparatively low. The mortality is lower in proportion to the population than most cities of its size. The death rate from tuberculosis, compared with the general death rate, is also surprisingly low in this city. Our climate is evidently maligned because theoretically it should be a powerful predisposing factor in its causation.. These conditions, so pleasant to contemplate, are certainly not due to special efforts intended to check the spread of an infectious disease, because no city of its size in this country has done so little in that direction. In the campaign against tuberculosis it has been a laggard, and smothering and evading facts will not help the censurable situation. We have a small percentage of the submerged. Buffalo is a city of homes and the population is spread over an unusually large area. The tenement house population is comparatively, still a slight and controlled factor. Foci of infection are scattered and easy to locate and control. Unsanitary homes and overcrowding, conditions which make an infected building hard to fight, are the exception here. Fresh air can still be obtained by the vast majority of people, if they have sense enough to desire that seeming necessity of life. Under all the circumstances there should be very little tuberculosis in Buffalo. The physical conditions are not favorable to its spread, and the field for a battle with the monster foe is technically strong. Let us examine the local situation.

During the year 1907, 554 deaths were reported as due to tuberculosis. Of this number 497 deaths were caused by tuberculosis of the lungs, and 57 from other forms of tuberculosis. All statistics of mortality are necessarily more or less inexact. There is opportunity for an error in diagnosis and an unknown number of false returns. Undoubtedly a certain percentage of deaths from tuberculosis are attributed to other diseases of the respiratory tract, particularly pneumonia. The belief is now widespread that the question of life insurance plays a part in causing false returns when tuberculosis happens, unfortunately, to be the cause of death. The actual number of those infected with tuberculosis cannot be known. Although tuberculosis is designated as an infectious disease only a small portion of the cases are reported to the Board of Health. We are compelled to estimate the number of tuberculosis victims by the usual method of multiplying the number of deaths by five. Thus we assume that there are 2,770 people fatally infected to maintain the ghastly number of deaths. If we include all infectious, surgical and medical in character, and could account for those who recover, the number would be much larger. Phillip believes that the number of deaths should be multiplied by at least ten. It seems fair to state that about one person in each 140 of the population of Buffalo is fatally infected with tuberculosis. It may be mentioned casually that these facts are in strange company with the slogan that tuberculosis is curable, preventable and unnecessary. There must be something wrong somewhere. Now the vast majority of consumptives are attacked during the years of greatest productivity. They succumb to a preventable disease when best prepared to struggle for existence and the welfare of those dependent. It is customary to estimate the loss of wealth to a community by illness and death due to the white plague. Several methods may be employed. They vary in the data included in technic and conclusions. However derived they are startling and are used to arouse the public and stimulate interest in questions of public health. The dose of statistics has been administered also to show that an expenditure to save human beings is a good investment, and also to justify some increased taxation in benighted regions where ideas of false economy prevail. Now the value of a human life cannot be estimated. The loss means one thing to the desolate and grief stricken family and another to the city or state.

There never has been a method devised to measure the incalculable value of character and its influence, though dwelling in the precincts of poverty, and failure to receive an education and a fair chance are inestimable. But from a cold, materialistic, commercial standpoint the average earning power, the loss of an expected duration of life and the cost of illness may form a basis for computing approximately, the loss of wealth caused by tuberculosis in Buffalo. The mean figure according to the method of Newsholme, Fisher, Phillip and Wilcox will be about $3,500,000 annually. A newspaper announcement that a new factory would be located in Buffalo whose employment would bring 500 workingmen to Buffalo might, perhaps, attract more attention. Would it not be wiser to join in an attempt to save a number of the 500 victims of tuberculosis who live here and would like to stay if they had a chance. I might dwell longer on the economic aspect of this subject if the belief had not grown strong that statistics used to stimulate interest and support will not win in this vast conflict. The people have become accustomed to the story of a terrible hidden scourge and the revelation must be brought closer so that they must see and feel. Appeals to sympathy, justice and fair play will accomplish more. If these are not successful then hard, rough facts must be driven home and clinched until the disgrace and shame cannot be escaped.

Exaggerated statements to show that the prevalency of tuberculosis has declined are sometimes employed to encourage a hopeful view or used to palliate the effects of stupid apathy. There is room for discussion when the causes ot an incontrovertible decrease in proportion to the population is analyzed. That it is due to special preventive measures I refuse to assent. The decline began twenty years before the cause was known and was apparently as rapid during that decade as it has been since. Radical efforts to combat tuberculosis have nev\r been employed but once. That was in Italy in the 15th Century. Suspicion aroused more through prevention than knowledge has accomplished since. This fact is not mentioned to recommend its repetition. It is extremely important at the present time to avoid the misleading conclusion that the actual number of deaths from tuberculosis has decreased. The proportion of deaths to the population has steadily declined but the number suffering from tuberculosis, and the number dying from that disease, is greater than ever before. There is more need of relief and succor because we claim to know how it can be accomplished. There is more intelligence, more people who can help, and more money if it can be reached. A few words concerning the remarkable regularity of the death rate in Buffalo seems necessary to reveal possible methods of attack. I am inclined to believe that the striking uniformity of the figures year after year can be partially explained.

The disease is distinctly endemic and largely due to infection in the home or workshop. To maintain the yearly average there must be four groups, one tottering on the edge of the grave, one advanced, one moderately advanced, and one freshly infected or incipient. For every death there must be one or more recently infected. The ghastly army moving slowly to the grave must be constantly joined by recruits, and the drafting of new victims must be performed with striking accuracy to keep the ranks full. The chance for infection is strangely limited. Immediate and prolonged contact seems to be necessary as a rule. The predisposing causes are not underestimated but the direct source of infection is often overlooked. A dark, overcrowded home is one thing, but insufficient food and poor ventilation with one consumptive in the building is a more important one. Examining those exposed is sure to be one of the most valuable methods we possess, when its importance is fully realised. When there is an advanced case look for an incipient. That is the place to find them and to learn how to detect them. If searched for they will be recognised in at least 20 per cent. of the homes of the poor. The advanced case must lead to the early one and the unwilling victim snatched from the horrid throng. Well what has been done in Buffalo to prevent disease and save life? Tuberculosis has never been attacked or treated as an infectious, curable disease. It must be only too obvious that the first step would be to locate the sources of infection and control them so far as possible. The consumptive must be found, his location known, and the extent of danger appreciated.

During the year 1907, 520 cases of tuberculosis were reported to the Board of Health, and there were 554 deaths from that disease. An additional number should be added where information was gained by bacteriological examination of sputum. Allow for that additional number and it will be found that in 1907 and 1908 less than one-third of the cases were recorded. Evidently the existence of a case becomes known by the report of a death, and no more will probably be heard from that home until another death occurs. There is fumigation after death and to an unknown extent the danger from further infection may be obviated. But the majority of consumptives communicate the disease before death alone eradicates the danger. This method of control is pursued in many cities and there are many reasons why Buffalo should not be subjected to unjust criticism. A pamphlet with ample instructions for the avoidance of infection has been prepared by the Health Department and all physicians can obtain it. But the Health Department is not supported properly by the medical profession and it can only move a few steps in advance without general co-operation. Under existing circumstances the people must depend upon the physician for information and guidance to secure protection.

Last year I questioned 100 patients in Buffalo who desired admission to Ray Brook, with the object of learning how many had received instructions from physicians concerning methods of prevention. Ninety per cent. of them were certainly expectorating bacilli, as only one out of 23 were incipient cases. Less than one-third had received instruction which could be termed at all adequate for the protection of the family or the public. In not a single instance had members of the family been examined to learn if others might be already stricken with a communicable disease. The results of such conditions are too apparent to call for any comment. Undoubtedly much is demanded of the physician by the public and many times arduous, disinterested work is unappreciated, but nothing draws the line so sharpely between commercialism, quackery, and the fads and cults as the unselfish endeavor to promote public health. Prevention can be made effective without inciting hysteria or phthisiophobia or adding hardship to affliction. One of the most hopeful features in the whole problem of tuberculosis is that the victim can be made harmless to others without irksome restraint. In any crusade against tuberculosis the need of hospital facilities for proper care and segregation are so vital that any argument seems unnecessary. Newsholme and others have shown conclusively that the death rate declines in proportion to the number of beds provided for special care. To the best of my knowledge Buffalo has the smallest number of beds for consumptives in proportion to its population than any city of its size in this country. Only sixty beds have been provided for years. They are located in an excellent building which constitutes a part of an almshouse. Boston has 770 and 850 more will be provided in the State of Massachusetts. New York City has 1,900 and is adding each year a large number. Chicago has begun the construction of a hospital for consumptives to cost one million dollars, aside from the large number of beds now furnished for consumptives. Buffalo and Erie County have provided a bed for each fifty consumptives. The victim must become a county pauper when he enters the alleged Erie County Hospital for Consumptives. No clever sophistry thus far invented will make him anything else. It is well-nigh impossible to obtain the consent of a patient to go there. There is an excellent medical staff in attendance. Effort after effort has been made to secure reforms, but thus far it has been defeated.

The one agency offered by the county and the city which pays about 85 per cent. of the taxes and is responsible for about 95 per cent of the patients is largely nulified by unfortunate and easily remedied conditions. Each year there is a new excuse for delay and the city seems powerless or too careless to act. Fortunately there are signs that the state will find a way to compel the proper provision for advanced cases and the clutch that has held so long will be made to yield.

The New York State Hospital at Ray Brook has been of great assistance to Buffalo until last summer, and all incipient cases applying for admission had been admitted. To be sure only a small percentage of such cases were detected for various reasons but there was, at least, a place to send them. Now the institution is filled and we are largely deprived of that opportunity for relief. The patient who applies today must wait about three months to gain admission. By that time only a small percentage can be allowed admission under the organic law and in accordance with the definition of the National Association for the Study and Prevention of Tuberculosis. Consequently no place is provided for the curable cases.

Thus far practically all scientific and progressive work in Buffalo has been inaugurated and maintained by private charity. The District Nursing Association by sending nurses and food to the poor sick have reached destitute consumptives who might otherwise have suffered from neglect and absence of any supervision. The Tuberculosis Dispensary established and supported by the Charity Organisation Society is performing most effective service and the influence is constantly extending. Relief is combined with investigation, control, education and continuous close touch with the patient and his home surroundings. Two of the most commendable features of the intelligent plan in operation are the opportunity offered and grasped to make an early diagnosis and teach the methods employed, and the examination of those exposed. The Trinity Class system working in conjunction with the dispensary supplies the best method of instruction and supervision at present known. The visits of the Dispensary Nurse and the lady in charge of the inspection of the class members completes as perfect a plan as can be devised under existing deplorable conditions. Two things must be remembered: these private agencies only touch a fringe, and while the work is usually quite effectual it is very often made incomplete, or almost nullified at times, by the lack of provision for hospital care.

The history, purpose and results of one summer's trial of a Day Camp will be presented by Dr. Eckel, who deserves high praise for its successful management. It might be mentioned that two of the chief objects sought in establishing the Day Camp was to demonstrate, if possible, that results could be obtained by proper methods in this climate and the need of a municipal hospital located in the vicinity of Buffalo. The results should silence objections that little or nothing could be accomplished in Buffalo. It has been revealed once more that method and discipline form a large part of the hygienic treatment of tuberculosis.

So much for the present conditions and the lack of facilities for a decided advance so marked at this time in other large cities. It is time to consider the question—What can be done?

The time has arrived when an association for the relief and control of tuberculosis can do effective work in Buffalo if adequately supported. Several organisations which have in the past attacked along various lines have joined interests and formed the nucleus for a large representative body, the objects of which are to secure registration and prevention. A municipal hospital and increased provision for advanced cases. The establishment and maintenance of dispensaries, class systems, day camps, increased nursing and visitation among poor consumptives and continued sane education of the public. The degree of accomplishment and the extent of its influence will depend upon a large membership and liberal contributions. The encouraging features which have made a campaign in Buffalo more promising are the immediate probability that registration of cases by physicians will be obtained by the enforcement of law, and the awakening to the lamentable lack of hospital facilities. A state law making registration compulsory was signed by the Governor last May. It is still inoperative in this city. The blanks containing the information desired have not as yet been furnished by the State Board of Health and the local health department is impatiently waiting for that material. Nevertheless the failure on the part of a physician to report a case of tuberculosis exposes him to prosecution and punishment. Health authorities can also be taken into court for failure to comply with the specifications. When the law becomes operative a question will arise as to the practical value of the mass of material aside from a statistical collection of facts. Not much will be gained if the number afflicted is known unless prevention is more thoroughly introduced. Obviously, the Health Department must utilise the information to check invasion, eliminate danger, and seize the opportunity for investigation and instruction. How this shall be done is a problem worthy of consideration by this Academy. This work, when undertaken, will require a new form of activity in the Health Department and lead to the inauguration of measures which have made New York City famous throughout the world. While experience and reflection makes one wary of a multitude of counsel one positive conviction grows stronger. Prevention will never be successfully employed unless it is associated with humane relief and institutional control for a constantly increasing percentage. It is difficult among the intelligent and ignorant; and impossible in the home of the incorrigible. The long period of exposure, and the numerous chances for a slip are often perplexing elements. It is absolutely successful in a properly managed special institution where precise methods, prolonged training and example lead to the formation of a habit. All cannot go to a hospital and many will not avail themselves of the chance, but the whole question must be settled by the percentage method. We must depend at present almost entirely upon the family physician.

I plead for a municipal hospital for incipient tuberculosis in the vicinity of Buffalo, and increased accommodation for the advanced victims. The need of an institution devoted to the care of the early case in now pressing and imperative. The physician is once more in the humiliating, powerless position of detecting incipient disease while he knows that there is no hope of prompt necessary succor. This condition cannot be tolerated. It is unspeakably brutal, and cruel, and distinctly barbarous from any aspect. The medical profession cannot afford to be silent or evade the consequences for a day. There is a critical emergency and it must be remedied by next spring. We have a unique anomaly in our system of charities by which a class die simply because they are poor. They are denied the benefits of medical knowledge and I tell you that the depicted results would form the saddest chapter in the history of the age.

Bountiful help flows to the other dependent sick, why not to the consumptive? They are outcasts and deliberately forgotten, and the people can still be happy while they struggle along for three and a half years before death closes the tragic scene. Shall we wait supinely in the hope of future partial relief and argue about the duty of the state and quaint theories concerning taxation which cannot be escaped? Shall the magnitude of the problem frighten and make us falter? It is vastly more colossal and difficult in New York City, but the men there move from one intrenchment to the other and the battle cry fills the air. Assume that the state should ultimatedly carry the burden of the care of incipient consumptives, how long must we wait for required benefits? If the institution at Ray Brook could be doubled in capacity by magic tonight it would be more than filled as soon as a proportion of those waiting could arrive within its portals. If the legislature, in its wisdom, should decide this winter to build more hospitals two years would elapse before they would be ready for occupancy.

One institution was established as a model, to teach proper care, demonstrate results, and encourage an early diagnosis. It required four years to fill 164 beds, and there are at least 16,000 incipient consumptives in the state. About 50 per cent. of the patients admitted could be classified as incipient victims. Of these more than 80 per cent. apparently recover, and allowing for the unfortunate conditions which promote relapse about 75 per cent. remain well five years after the date of discharge. The percentage of recoveries in truly incipient cases is almost impossible to determine because they are rarely recognised and correctly advised. The ultimate results and the lasting recoveries will depend largely upon a new form of interest addressed to the patient after discharge. It is time that the method of continual supervision after discharge from a hospital should be made more effective here. There is a strange tendency upon the part of some physicians, while urging the importance of prevention, which is now largely a delusion, to forget that the saving of life is one of the ideal forms. An early diagnosis and prompt relief ensures prevention when the danger is slight or *nil*, shortens the term when it is necessary, obviates progeny, and at least secures education if the result of treatment should prove disappointing.

The hasty remark is sometimes made that the consumptives are not treated any better than cattle. This is a careless statement. The infected animal is killed to protect others in the herd. Cattle have a palpable commercial value. The state pays the owner two-thirds of the value of the property destroyed. The whole proceeding touches the farmer's pocket and influences his vote. The human being is not protected from infection. He is allowed a lingering illness undisturbed. The bereaved receives no compensation. The United States Government can be tremendously active when an epidemic of foot and mouth disease is threatening. Thus far the epoch-making meetings of the governors to consider the waste of natural resources have not been marked by any discussion of the loss of life by preventable illness. Yet some humble citizens cling to the idea that the health and welfare of the workers constitute the chief source of natural wealth. Sometime the people will insist upon protection from disease as they do upon protection from crime and fire, but they will have to pay for it.

Some way must be provided to increase provision for the care and segregation of advanced cases in this city. Not alone to diminish infection but to make the gradual decline as easy as possible in accordance with the simple dictates of humanity. The place where relief could be given and accommodation extended indefinitely is at the so-called Erie County Hospital. The increase in the number of beds is now too slow. There is no time to wait for the erection of another almshouse elsewhere. The need calls for immediate action. Ultimately the institution should become a municipal hospital. Any controversy relating to the policy of caring for the incipient or the advanced case seems entirely unnecessary. One plan must supplement the other. It is true that the advanced case in about 50 per cent. of instances is simply the stupidly neglected early victim. He is the result of a sociological blunder but the price of wretched inhumanity must be paid. If we fail to find the consumptive early and reach him with rational help the penalty must be paid by protecting the public and helping the innocent sufferer until he is dead.

Finally, it may be asked, can consumption be conquered in the near future and the wise will not prophesy. A big sociological problem is involved and no one can predict how long the people will slumber. There is sufficient knowledge to wage a most successful campaign, and there can be but one answer to the demand that the unfortunate consumptive shall be treated like a sick human being. The timid and conservative individual is no longer needed in the fight. The place for him is in the rear where his indecisions and whining complaints can be indulged with impunity. If he believes that civilisation will permit the ravages of tuberculosis to continue without a battle let him hug the delusion now and enjoy his antiquated views while he can because the worst days in the conflict have passed and determined, unrelenting progress has begun all along the lines. The essential need now is money, money, money. It must be wrung from the private purse, the state, the county and the city, and those who have worked and waited for years no longer doubt the outcome.

We have a Governor in this state with remarkable breadth of view and sympathies, keen enough to fathom the depths of cruel misfortune, and he has iron in his blood. The other day he used these words in a clarion call which will ring and echo through the state and arouse the true citizen to action and renewed effort.

With 5,400 deaths yearly outside of Greater New York, it appears that there are only 250 beds for tuberculosis patients. Economy is necessary, but while we are careful and prudent with regard to new undertakings it would hardly seem that we could close our eyes to an obvious duty. Should not private purses be opened in order that suitable provision be made for the poor struck down, not by their own fault, but because of an exposure from which they should have been protected by an enlightened community. Every death from tuberculosis may be said to be a stain upon the honor of the state. Certain it is to the extent to which it might have been prevented by proper precautions against infection.

These quoted remarks apply with singular forcefulness to Buffalo. The stain is large and hideously plain to those who will not refuse to see. Tonight we have exposed some of the ragged edges.

26 Linwood Avenue.

